# Plant Growth under Natural Light Conditions Provides Highly Flexible Short-Term Acclimation Properties toward High Light Stress

**DOI:** 10.3389/fpls.2017.00681

**Published:** 2017-05-03

**Authors:** Tobias Schumann, Suman Paul, Michael Melzer, Peter Dörmann, Peter Jahns

**Affiliations:** ^1^Plant Biochemistry, Heinrich-Heine-University DüsseldorfDüsseldorf, Germany; ^2^Department of Plant Physiology, Umeå UniversityUmeå, Sweden; ^3^Physiology and Cell Biology, Leibniz Institute of Plant Genetics and Crop Plant Research (IPK)Seeland, Germany; ^4^Molecular Biotechnology/Biochemistry, Institute of Molecular Physiology and Biotechnology of Plants (IMBIO), Rheinische Friedrich-Wilhelms-University BonnBonn, Germany

**Keywords:** light acclimation, membrane dynamics, non-photochemical quenching, photooxidative stress, photosynthesis, photoprotection, thylakoid membrane

## Abstract

Efficient acclimation to different growth light intensities is essential for plant fitness. So far, most studies on light acclimation have been conducted with plants grown under different constant light regimes, but more recent work indicated that acclimation to fluctuating light or field conditions may result in different physiological properties of plants. Thale cress (*Arabidopsis thaliana*) was grown under three different constant light intensities (LL: 25 μmol photons m^−2^ s^−1^; NL: 100 μmol photons m^−2^ s^−^^1^; HL: 500 μmol photons m^−2^ s^−1^) and under natural fluctuating light (NatL) conditions. We performed a thorough characterization of the morphological, physiological, and biochemical properties focusing on photo-protective mechanisms. Our analyses corroborated the known properties of LL, NL, and HL plants. NatL plants, however, were found to combine characteristics of both LL and HL grown plants, leading to efficient and unique light utilization capacities. Strikingly, the high energy dissipation capacity of NatL plants correlated with increased dynamics of thylakoid membrane reorganization upon short-term acclimation to excess light. We conclude that the thylakoid membrane organization and particularly the light-dependent and reversible unstacking of grana membranes likely represent key factors that provide the basis for the high acclimation capacity of NatL grown plants to rapidly changing light intensities.

## Introduction

Efficient acclimation to changing environmental conditions is a prerequisite for the survival and competitiveness of plants in the field. Proper acclimation to the light availability at a given habitat is essential to allow for efficient light utilization under light-limiting conditions and to avoid photo-oxidative damage under excess-light condition. Long-term acclimation to either low light (LL) or high light (HL) conditions occurs in the time range of days to months and involves—among others—adjustments of leaf architecture, chloroplast structure, composition of the photosynthetic electron transport chain, and regulation of photosynthetic light utilization (Boardman, 1977; Anderson, 1986; Schoettler and Toth, 2014). Typical characteristics of HL (or sun) acclimated plants in comparison with LL (or shade) acclimated plants are:

1. Increased thickness of leaves with more cell layers and larger cells (Björkman and Holmgren, 1963; Ludlow and Wilson, 1971; Wild and Wolf, 1980; Weston et al., 2000).
2. Increased number of chloroplasts per cell (Anderson et al., 1973; Anderson, 1986) with reduced grana stacking (Anderson et al., 1973; Lichtenthaler et al., 1981).
3. Higher Chl a/b ratio (Boardman, 1977; Wild, 1980; Lichtenthaler et al., 1981; Bailey et al., 2004) and increased β-carotene and xanthophyll cycle pigment levels (Anderson, 1986; Bailey et al., 2004).
4. Higher photosystem II (PSII)/PSI ratio and smaller PSII antenna size (Schoettler and Toth, 2014; Albanese et al., 2016).
5. Higher electron transport rates, higher CO_2_ assimilation rates and higher light compensation points (Björkman and Holmgren, 1963; Ludlow and Wilson, 1971; Boardman, 1977; Wild, 1980).
6. Higher energy dissipation capacity (Brugnoli et al., 1994; Demmig-Adams and Adams, 1996; Park et al., 1996; Ballottari et al., 2007; Mishra et al., 2012).

These characteristics apply to extreme sun and shade plants in the field, to sun, and shade leaves of the same individual plant in the field and to plants grown under different controlled light conditions in the lab.

In contrast to other environmental factors, the light intensity may vary in the short-term (seconds to minutes) by orders of magnitudes in an unpredictable manner such as on cloudy days. Particularly, plants at normally shady sites which are frequently exposed to HL, must properly adjust the photosynthetic capacity to overcome the challenges related to photo-oxidative damage under such fluctuating light conditions (Li et al., 2009). The fastest photoprotective response of plants and algae to rapidly increasing light intensities is the non-photochemical quenching (NPQ) of excess light energy in the antenna of photosystem II (PSII) (Müller et al., 2001; Jahns and Holzwarth, 2012; Ruban et al., 2012; Derks et al., 2015; Goss and Lepetit, 2015). Among the different components that contribute to the overall NPQ (Quick and Stitt, 1989; Walters and Horton, 1991; Nilkens et al., 2010), the pH-regulated qE component represents the main constituent of NPQ under most conditions and is the fastest (within minutes) inducible and relaxing component (Nilkens et al., 2010). In land plants, qE is strictly regulated by the thylakoid lumen pH (Krause et al., 1982) and requires the PsbS protein for rapid activation (Li et al., 2000). PsbS acts as a sensor of the lumen pH (Li et al., 2004) and is supposed to activate qE by conformational changes in PSII antenna proteins (Horton et al., 2005) through the interaction with LHCII complexes (Correa-Galvis et al., 2016; Sacharz et al., 2017).

Apart from this central function of PsbS, qE is also regulated by the xanthophyll zeaxanthin (Zx) (Demmig et al., 1987; Horton et al., 1996, 2005; Nilkens et al., 2010), which is formed in high light in the de-epoxidation reactions of the xanthophyll cycle from violaxanthin (Vx) (Jahns et al., 2009). The function of Zx in NPQ, however, is not only limited to the pH-regulated qE mechanism, but Zx is also involved in more slowly relaxing NPQ states such as qZ (Dall'Osto et al., 2005; Nilkens et al., 2010) and qI (Adams et al., 2002; Demmig-Adams et al., 2006; Nilkens et al., 2010). In particular, the close kinetic correlation of Zx epoxidation and the recovery from photoinhibition (Jahns and Miehe, 1996; Verhoeven et al., 1996; Reinhold et al., 2008) supports a critical role of Zx in qI. This function is likely related to the sustained down-regulation of PSII, which has been observed along with the inactivation of Zx epoxidation in overwintering evergreen plants (Adams et al., 1995a,b; Ebbert et al., 2005; Zarter et al., 2006b).

Though activation and/or maintenance of different NPQ states are correlated with the presence of Zx, this does not allow any conclusions about a specific function of Zx in energy quenching (Jahns and Holzwarth, 2012). However, a direct function of Zx in qE in minor antenna complexes has been derived from transient absorption measurements performed with intact thylakoids (Holt et al., 2005) or isolated PSII antenna complexes (Ahn et al., 2008; Avenson et al., 2008). In contrast, an indirect function of Zx in trimeric light-harvesting complexes of PSII (LHCII) has been proposed on the basis of resonance Raman spectroscopy (Robert et al., 2004; Ruban et al., 2007). These contrasting observations imply that different quenching mechanisms and/or quenching sites with different roles of Zx contribute to NPQ. In fact, time-resolved Chl fluorescence measurements support the view that at least two different quenching sites/mechanisms are active in diatoms (Miloslavina et al., 2009), green algae (Amarnath et al., 2012) and vascular plants (Holzwarth et al., 2009). Measurements with intact leaves of Arabidopsis wild type and NPQ mutant plants identified two different quenching sites, termed Q1 and Q2, with different requirements for PsbS (involved in the activation of Q1) and Zx (required for activation of Q2; Holzwarth et al., 2009).

The increased NPQ capacity of high-light acclimated plants (Brugnoli et al., 1994; Demmig-Adams and Adams, 1996; Park et al., 1996; Ballottari et al., 2007; Mishra et al., 2012) is typically accompanied by the accumulation of higher levels of PsbS and Zx than under LL (Demmig-Adams et al., 2006; Zarter et al., 2006a; Albanese et al., 2016). This underlines again the essential role of these two factors for NPQ. Recent work has shown that field-grown plants acclimated to natural HL conditions develop a higher NPQ capacity compared to plants grown under constant HL conditions in the lab (Mishra et al., 2012) and high NPQ capacities have been observed in evergreen plants acclimated to HL during winter (Demmig-Adams et al., 2006). Such high quenching capacities in evergreen plants have been correlated with a light-induced partial unstacking of the thylakoid membrane (Demmig-Adams et al., 2015). Interestingly, super-quenching states in the dinoflagellate *Symbiodinium* have recently been shown to be related to the activation of an energy spill-over mechanism of quenching (i.e., efficient energy transfer from PSII to PSI), which is also accompanied by structural rearrangement of the thylakoid membrane (Slavov et al., 2016).

In this work, we characterized the acclimation of Arabidopsis plants to different constant light intensities in comparison with plants grown under natural fluctuating light (NatL) conditions. We hypothesize that growing plants under fluctuating light might provide a better adaptation of the plants to high light stress. Analysis of the morphological, physiological, and biochemical characteristics indicated that NatL plants combine properties of LL and HL acclimated plants. NatL plants exhibited a high NPQ capacity among all plants grown at the different light regimes. Time-resolved Chl fluorescence analysis showed that this high NPQ capacity of NatL plants is based on an efficient qE quenching whose activation is accompanied by reversible changes in the thylakoid membrane stacking.

## Materials and methods

### Plant growth

*Arabidopsis thaliana* (ecotype Col-0) plants were cultivated on soil (BP substrate, Klasmann-Deilmann GmbH, Geerste, Germany) under long day conditions (14 h light/10 h dark) at 20°C and three different light intensities: Low light (LL, 25 μmol photons m^−2^ s^−1^); normal light (NL, 100 μmol photons m^−2^ s^−1^) and high light (HL, 500 μmol photons m^−2^ s^−1^). LL and HL plants were transferred into the respective light regime after 2 weeks of growth under NL conditions. Plants grown under natural light (NatL) conditions were transferred to an east-facing balcony outside of the lab (Düsseldorf, Germany, 51°11′18.5″N 6°48′00.5″E). Plants were watered manually, because the site was sheltered from rain. Full sunlight exposure was only possible before noon due to shading of the plants by surrounding buildings. The daily photoperiod varied between 14 and 16 h. The median light intensity received by NatL plants was about 150 μmol photons m^−2^ s^−1^, with a 95% quantile of 1230 μmol photons m^−2^ s^−1^ at its upper range (see Figure S1). For all experiments, about 5 weeks old plants were used for NL, HL, and NatL conditions, and about 6 weeks old plants for LL conditions.

### Pigment analysis

Intact leaves or leaf discs were harvested and immediately shock frozen in liquid N_2_. After pestling, pigments were extracted with 1 ml of 100% acetone. After short centrifugation, samples were filtered through a 0.2 μm membrane filter (GE Healthcare, Buckinghamshire, UK) and stored at −20°C until analysis. Pigments were separated and quantified by HPLC analysis as described (Färber et al., 1997).

### Isolation of chloroplasts and thylakoid membranes

Intact chloroplast were prepared according to Kley et al. (2010). In brief, 2–5 grams of dark-adapted leaves were kept for 2 h at 4°C and then homogenized in 25 ml of isolation medium (0.3 M sorbitol, 20 mM Hepes/KOH pH 7.6, 1 mM MgCl_2_, 1 mM MnCl_2_, 5 mM EDTA, 5 mM EGTA, 10 mM NaHCO_3_) supplemented with 0.1% (w/v) BSA and 330 mg/l Na-ascorbate. The homogenate was gently filtered through one layer 50 μm Petex polyester mesh (Sefar, Thal, Switzerland) and then loaded on a Percoll cushion [50% (v/v) Percoll in isolation medium]. After centrifugation for 10 min at 4°C and 2000 × g, the resulting pellet, which contained intact chloroplasts, was gently resuspended in isolation buffer. The chloroplast suspension was centrifuged for 5 min at 4°C and 2,000 × g and finally resuspended in a small volume (100–250 μl) of isolation buffer. Thylakoid membranes were isolated from chloroplasts after osmotic shock with 5 mM MgCl_2_.

### Determination of the Chl content of chloroplasts

Fifty microliters of four dilutions (1:10, 1:20, 1:50, and 1:100) of isolated intact chloroplasts were transferred to a Neubauer counting chamber and the number of chloroplasts was quantified via counting 4 out of 16 squares of the counting chamber. The Chl content per chloroplast was calculated on basis of the Chl concentration of each dilution.

### SDS-PAGE and western blot analysis

SDS-PAGE was performed according to Laemmli (1970). 13.5% acrylamide gels were used and 8–20 μg total protein were loaded on the gel for each sample. Proteins were transferred to a PVDF membrane (BIORAD, Hercules, USA) using a discontinuous blotting system according to Kyhse-Andersen (1984). Coomassie and Ponceau S staining of gels and membranes, respectively, were used as loading and transfer controls. Anti-PsbS (1:8000, commissioned work by Pineda Antikörper Service, Berlin, Germany) was used as antibody. The second antibody (1:10000, anti-rabbit-IgG, Sigma-Aldrich) was detected by chemiluminescence (PicoLucent™, GBiosciences, St. Louis, USA). Chemiluminescence was detected using the LAS-4000 mini (Fujifilm, Tokyo, Japan). Band intensity was quantified using the freeware Image Studio Lite (LI-COR Biosciences, Lincoln, USA).

### Spectroscopic determination of PSI, PSII, and Cyt b_6_f

For the determination of the PSI content, isolated thylakoids equivalent to 50 μmol Chl were suspended in 1.5 ml measuring medium [0.2% (w/v) n-dodecyl-β-D-maltoside, 30 mM KCl, 10 mM MgCl_2_, and 30 mM Hepes/KOH, pH 7.6]. After short centrifugation (45 s, 10,000 × g), 1.2 ml of the supernatant was transferred into a disposable polystyrene cuvette (Sarstedt, Nümbrecht, Germany). 10 mM Na ascorbate and 100 mM methyl viologen were added to the sample and mixed carefully before the measurement. PSI was quantified using the P700 emitter/detector unit of a DUAL-PAM 100 (Walz, Effeltrich, Germany). Only fully dark-adapted samples were measured. Precautions were made that no trembling of the cuvette or the cuvette holder disturbed the sensitive measurement. After calibrating the P700 signal, a 200 ms saturation pulse was applied to the sample and the maximum amplitude of the signal was quantified. The dark baseline resembles the PSI in a fully reduced state, whereas at the maximal amplitude, PSI is in a completely oxidized state. PSI content was calculated as follows: Δc = ΔI/I/(2.3 × ε × d), with ε = 2.53 cm^2^ μmol^−1^, specific for the Dual-PAM system used for the experiments.

The amounts of PSII and cytochrome (Cyt) b_6_f were calculated from differential spectra measured with a photometer in a range of 540–575 nm. In this approach, absorption changes of Cyt b_6_, Cyt f, Cyt_559_, and Cyt_550_ were measured at different oxidation states (see below). The differential spectra were fitted against reference spectra and the amount of cytochrome b_6_f and PSII (Cyt_550_) was calculated.

Isolated thylakoids equivalent to 50 μmol of Chl were incubated for 10 min in the measuring medium [0.02% (w/v) n-dodecyl-β-D-maltoside, 30 mM KCl, 0.1 mM EDTA, and 30 mM Hepes/KOH, pH 7.6] to ensure complete grana unstacking. After blanking, 1 mM potassium ferricyanide was added to fully oxidize the cytochromes. After 1 min of incubation the spectra were recorded (10 cycles). Subsequently, 10 mM Na ascorbate was added to partially reduce the cytochromes. Samples were incubated for 5 min and spectra were recorded again in the range of 540–575 nm (10 cycles). Finally, to fully reduce all cytochromes, a spatula tip of dithionite was added and the cuvette was sealed with paraffin oil (150 μl) to prevent reoxidation of the cytochromes by aerial oxygen. After 8 min of incubation on ice, the spectra were measured (10 cycles). Averages from all cycles of each treatment were used for calculating the amount of PSII (Cyt_550_) and Cyt b_6_f.

### NPQ measurements

Steady state Chl fluorescence was measured with the DUAL-PAM 100 (Walz, Effeltrich, Germany). Dark-adapted leaves were illuminated for 30 min at the respective actinic light intensity, followed by 30 min dark relaxation. Saturation pulses (200 ms, 4,000 μmol photons m^−2^ s^−1^) were applied to determine the NPQ as (Fm/Fm′ − 1) (Krause and Jahns, 2004). Electron transport rates were estimated according to Genty et al. (1989). The redox state of Q_*A*_ was derived from the parameter qL = (Fm′− F)/(Fm′ − ${\text{F}}_{0}^{\prime }$) × ${\text{F}}_{0}^{\prime }$/F according to Kramer et al. (2004). The transient NPQ was determined from fluorescence measurements during 10 min illumination at 53 μmol photons m^−2^ s^−1^ (for NL, HL, and NatL plants) or at 13 μmol photons m^−2^ s^−1^ (for LL plants).

### P700 oxidation state

The redox state of P700 was determined with the DUAL-PAM-100 (Walz, Effeltrich, Germany) employing the saturation pulse method (Klughammer and Schreiber, 1994). In brief, leaves were illuminated at different light intensities in the range from 20 to 1,950 μmol photons m^−2^ s^−1^ and P700 absorbance changes were measured at 830 nm after 2 min of illumination at each light intensity. The P700 oxidation state was derived from the fraction of donor-side limited closed centers P700^+^ A, Y(ND).

### OJIP transients

Chl fluorescence induction transients (Stirbet and Govindjee, 2011) were measured with a Handy PEA fluorometer (Hansatech Instruments, Norfolk, UK). Dark acclimated leaves were illuminated for 1 s with 3,500 μmol photons m^−2^ s^−1^ at a gain multiplication of 0.5. The nomenclature of this measurement O-J-I-P resembles the different fluorescence states, with *O* = origin, ground fluorescence (F_0_); J, and *I* = intermediate states based on the reduction of Q_A_ (O-J phase) and the electron transfer to the PQ pool (J-I phase); *P* = peak, maximum fluorescence (Fm).

### Ultrafast fluorescence kinetics

Ultrafast lifetime measurements were carried out as described (Holzwarth et al., 2009) with detached leaves held in a rotating cuvette, front-face excitation of the upper side of the leaf, using laser pulses of 663 nm and a repetition rate of 4 MHz. F_max_ measurements were performed with dark-acclimated leaves infiltrated with 3-(3,4-dichlorophenyl)-1,1-dimethylurea. F_NPQ_ measurements were started after 30 min pre-illumination at 600 μmol photons m^−2^ s^−1^ using a mixture of red and amber light-emitting diodes. Kinetic data analysis and kinetic compartment modeling were performed as described (Holzwarth et al., 2009; Slavov et al., 2016).

### Light microscopy

Leaf material was fixed and prepared as specified in Table 1. Semi thin (2 μm) leaf cross sections were cut with a microtome (Leica Ultracut, Leica Microsystems, Bensheim, Germany) and leaf cross sections were stained for 2 min at 60°C with 1% (v/v) methylene blue, 1% (v/v) azur II in a 1% (v/v) aqueous borax solution. After washing and drying, cross sections were examined using a Zeiss Axiocam camera in in a Zeiss Axiovert 135 microscope (Zeiss, Oberkochen, Germany).

**Table 1:** **Sample preparation for transmission electron microscopy**.

**Microwave processing in a PELCO Bio Wave^**®**^34700-230 (Ted Pella, Inc., Redding CA, USA)**				
**Process**	**Reagent**	**Power [W]**	**Time [sec]**	**Vacuum [mm Hg]**
1. Primary fixation	2.0% (v/v) glutaraldehyde and 2.0% (v/v) paraformaldehyde in 0.05 M cacodylate buffer (pH 7.3).	0	60	0
		150	60	0
		0	60	0
		150	60	0
2. Wash	1 × 0.05 M cacodylate buffer (pH 7.3) and 2x aqua dest.	150	45	0
3. Secondary fixation	1% (v/v) osmiumtetroxide in aqua dest.	0	60	15
		80	120	15
		0	60	15
		80	120	15
4. Wash	3 × aqua dest.	150	45	0
5. Dehydration	Acetone series:30%, 40%, 50%, 60%, 70%, 80%, 90%, 1 × 100%. 1 × Propylenoxide.	150	45	0
6. Resin infiltration	Spurr's resin in propylenoxide: 25, 50, 75, and 100%.	250	180	5
7. Resin infiltration	100% Spurr's resin at RT on shaker for 16 h.			
8. Polymerisation	24 h at 70°C in flat embedding molds in a heating cabinet.			

*Protocol for combined conventional and microwave-proceeded fixation, dehydration and resin embedding of Arabidopsis rosette leaf tissue for histological and ultrastructural analysis*.

### Transmission electron microscopy

Transmission electron microscopy images were obtained with a FEI Tecnai Sphera G2 (FEI, Hillsboro, Oregon, USA) microscope. For comparative histological and ultrastructural analysis, microwave proceeded fixation, substitution and resin embedding of rosette leaves was performed as specified in Table 1. Sectioning and microscopy analysis was carried out as described previously (Daghma et al., 2011).

### Lipid analysis

Total lipids were extracted from 200 mg of leaves with chloroform/methanol. Harvested leaf material was immediately transferred into glass vials containing boiling water and boiled for 20 min to inhibit all lipase activity. After transferring the leaves into a fresh glass vial, 1 volume of chloroform:methanol (2:1) was added and samples were gently mixed. The green supernatant was transferred into a fresh glass vial and the leaf material was washed in a second step with 1 volume chloroform:methanol (1:2). The green supernatants were pooled and stored in a glass vial with Teflon® cap at −20°C. The leaf material was dried overnight in a drying chamber at 70°C and the dry weight was determined. Membrane phospholipids and glycolipids were quantified by direct infusion nanospray mass spectrometry on an Agilent 6530 quadrupole time-of-flight instrument (Gasulla et al., 2013).

### Gas exchange measurements

CO_2_ assimilation rates and light compensation points were derived from light response curves determined by gas exchange measurements [LI-COR-6400XT (LI-COR, Nebraska, USA)] under controlled CO_2_ conditions (400 ppm CO_2_, flow rate 300 μmol s^−1^, 102.4 kPa) at 20°C. Before each measurement, plants were light-acclimated for 15 min at 500 μmol photons m^−2^ s^−1^. Light response curves were measured from the lowest (25 μmol photons m^−2^ s^−1^) to the highest (2,000 μmol photons m^−2^ s^−1^) light intensity. Leaves were acclimated to the respective light intensity for 3 min. For determination of the maximum assimilation rate (P_max_) and the light compensation point (LCP), curves were fitted with Prism® applying a single exponential function.

### Statistical analysis

Differences among the analyzed variables under the different growth light regimes were evaluated statistically using Sigma Plot 12.5. For each variable, significant differences among growth light conditions were determined by ANOVA or—when neither the error normality nor the variance homogeneity criteria were fulfilled—by the Kruskall Wallis test. Subsequently, specific differences between light growth regimes were evaluated by the Holm-Sidak test (in the case of ANOVA) or by the Dunn's test (in case of the Kruskall Wallis test). Significant differences (*p* < 0.05) are indicated.

## Results

### NatL plants exhibit higher light use efficiency than plants grown under continuous light

**Figure 1:**
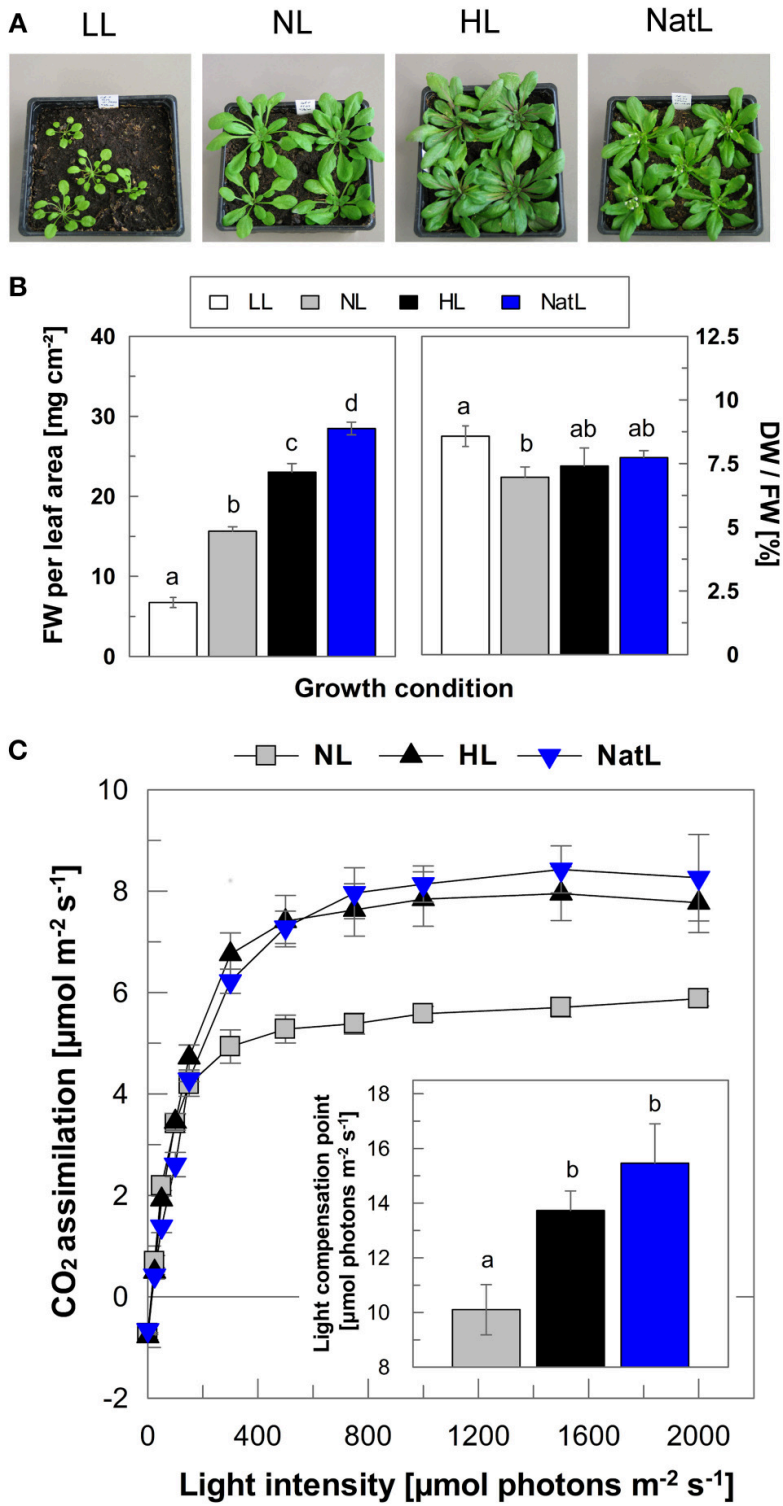
**Plant growth and CO_**2**_ assimilation rates. (A)** Typical phenotype of 6 weeks-old plants. Please note that 6 weeks-old plants are shown here to illustrate the differences in development, only. All analyses have been performed with about 5 weeks-old plants, and thus before onset of flowering. **(B)** Fresh weight (FW) of leaves in mg cm^−2^ and dry weight (DW) per FW in% (100 × DW/FW). Mean ± SE of six independent samples are shown. Significant differences (Holm-Sidak test, *p* = 0.05) are indicated. **(C)** Light response curves of NL, HL, and NatL grown plants. Insert, light compensation point derived from exponential fits of the assimilation curves. Mean values ± SE of 4–6 independent measurements are shown. Significant differences (Holm-Sidak test, *p* < 0.05) are indicated. Due to their small size, LL plants could not be measured.

Plants grown under constant high light conditions showed increased growth compared to those grown under constant low light as judged from the phenotype of 6 week-old plants (Figure 1A). The size of NatL plants was similar to that of HL plants. However, in contrast to all other plants, NatL plants already developed flowers after 6 weeks (Figure 1A), indicating that NatL conditions triggered a faster plant development. This was supported by analysis of the fresh weight (FW) per cm^2^ leaf area, which increased from about 10 mg in LL plants to 25 mg in HL plants, whereas NatL plants showed highest values of about 30 mg (Figure 1B). The dry weight (DW)/FW ratio showed no pronounced differences among the different plants (Figure 1B), reflecting that the DW showed similar relative differences among the different plants as observed for the FW. Moreover, NatL plants displayed high rates of net photosynthesis similar to HL plants (Figure 1C). The median light intensity under NatL conditions (about 150 μmol photons m^−2^ s^−1^) was about 30% of that under HL conditions (500 μmol photons m^−2^ s^−1^), but the corresponding difference in biomass was clearly much lower. We therefore conclude that plants grown under NatL have higher light use efficiency than HL grown plants.

### Leaf morphology of NatL plants is similar to that of HL plants

**Figure 2:**
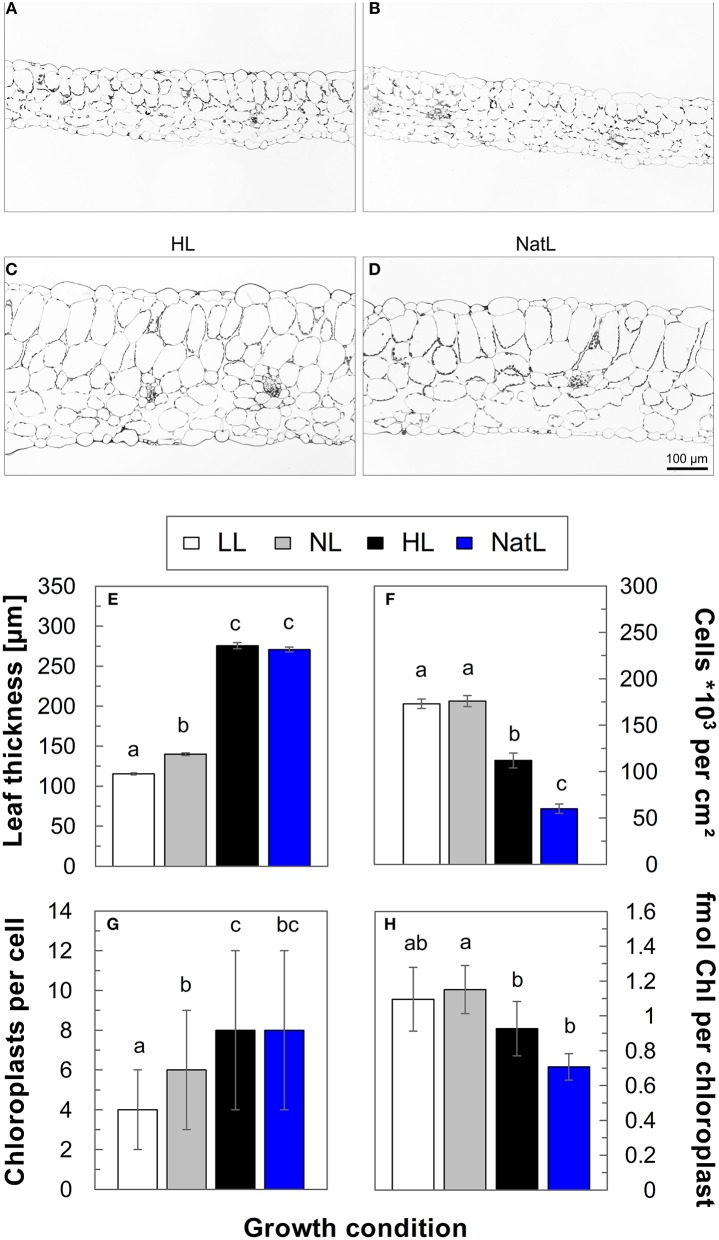
**Light microscopic analysis of leaf cross-sections**. Upper panel: Light microscopic images of leaf cross-sections from **(A)** LL plants, **(B)** NL plants, **(C)** HL plants, and **(D)** NatL plants. Lower panel: Quantitative analysis of **(E)** leaf thickness, **(F)** the number of cells per cm^2^ leaf area, **(G)** the number of chloroplasts per cell and **(H)** the number of Chl per chloroplast. Significant differences (Dunn's test, *p* < 0.05) are indicated. Data represent mean values ± SE of at least 116 leaf cross-sections in **(E)**, of at least 32 leaf cross-section in **(F)**, of cells from at least 6 images in **(G)**, and of at least 3 independent chloroplast preparations in **(H)**.

Microscopic analysis of leaf cross sections (Figures 2A–D) revealed a similar leaf thickness of about 115–130 μm in LL and NL plants, while growth under HL and NatL resulted in about 2-fold thicker leaves of about 270–280 μm (Figure 2E). The increased leaf thickness of HL and NatL plants was mainly due to elongated parenchyma cells (Figure 2F) and only partly related to an increased number of cell layers, which varied between 6 layers in LL plants, 7 layers in NL, and NatL plants, and 8 layers in HL plants. The number of chloroplasts per mesophyll cell increased from about 4 in LL plants to 6 in NL plants and 8 in HL and NatL plants (Figure 2G). An only slight difference was determined for the Chl content of chloroplasts (Figure 2H), which tended to decrease with increased growth light intensities, and was lowest in NatL grown plants.

### Thylakoid membranes of NatL plants share structural properties of LL and HL plants

**Figure 3:**
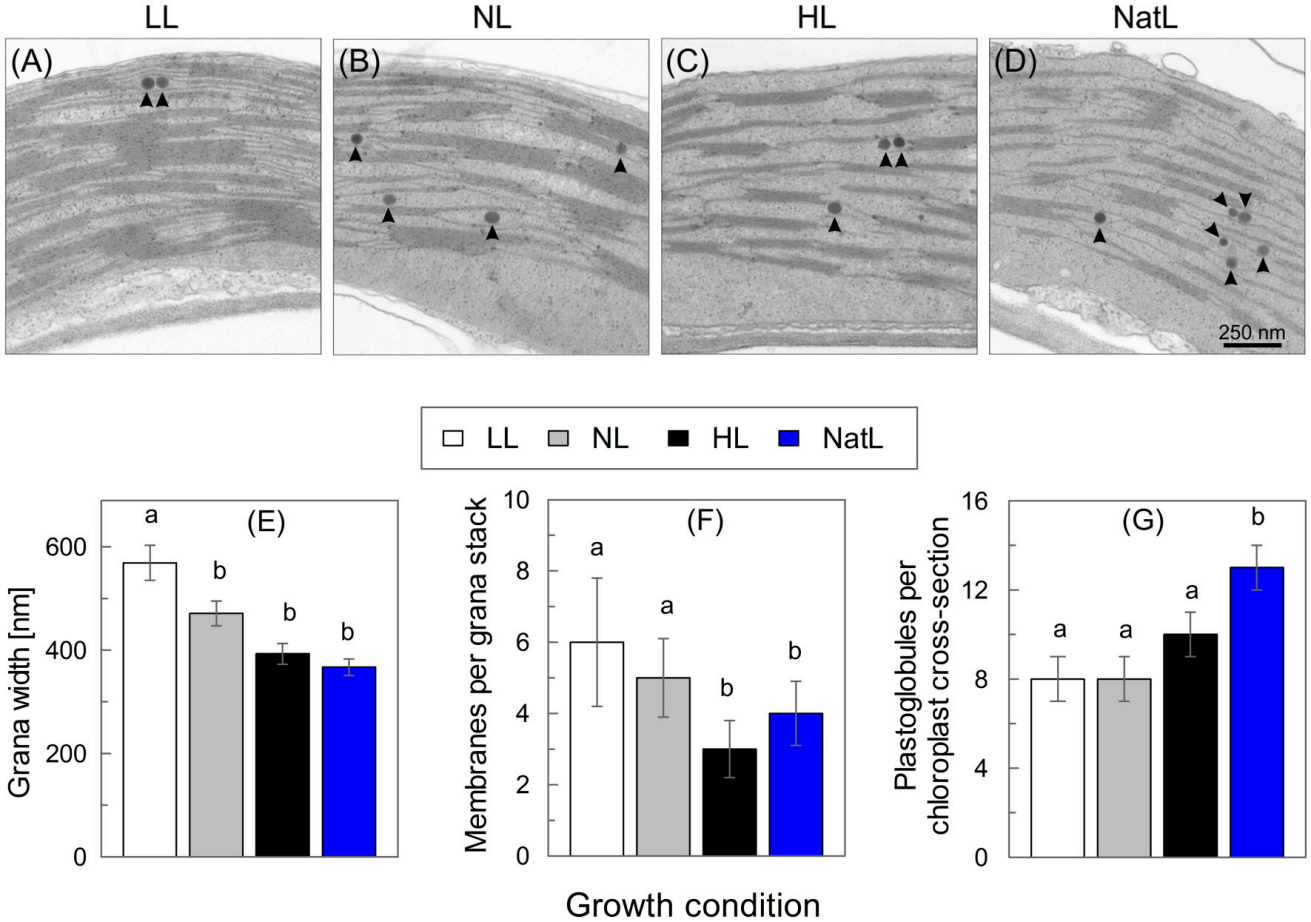
**Transmission electron microscopy analysis of chloroplasts**. Upper panel: Electron microscopic images of the thylakoid membrane structure of **(A)** LL plants, **(B)** NL plants, **(C)**, HL plants, and **(D)** NatL plants. Round dark structures (arrow heads) represent plastoglobules. Lower panel: Quantification of **(E)** the width of grana stacks, **(F)** the number of membranes per grana stack and **(G)** the number of plastoglobules. Mean values ± SE of at least six images are shown. Significant differences (Dunn's test, *p* < 0.05) are indicated.

The thylakoid membrane organization was investigated by transmission electron microscopy (Figures 3A–D). Chloroplasts from LL plants showed the highest density of thylakoid membranes in comparison to those from other growth conditions. In general, more and thicker grana stacks were detectable, which were connected by a large number of stroma lamellae (Figure 3A). On average, 6 membranes per grana stack and a grana width of about 570 nm were found for LL plants (Figures 3E,F). The overall thylakoid structure of NL plants (Figure 3B) was similar to that of LL plants, but the number of membranes per grana stack was reduced to about 5 and the grana width to about 470 nm (Figures 3E,F).

In contrast to LL and NL plants, the overall amount of thylakoid membranes and the overall degree of grana stacking was strongly reduced in HL plants, so that the relative fraction of stroma exposed membranes increased (Figure 3C). Grana stacks in HL plants typically consisted of only 3 membranes and the grana width was reduced to about 390 nm (Figures 3E,F). In NatL plants (Figure 3D), the thylakoid membrane system was similar to that in HL plants, but the number of membranes per grana stacks was significantly higher (4 membranes per granum, Figure 3F). However, in contrast to all other plants, NatL grown plants showed both, thin grana of 2 or 3 membranes as in HL plants but also some thicker grana with more than 6 membranes within the same chloroplast. This indicates that the thylakoid membrane organization in NatL plants shares properties of LL and HL acclimated chloroplast.

Strikingly, also the number of plastoglobules per chloroplast varied among the plants from different growth conditions (Figure 3G). While about 8 plastoglobules per chloroplast cross-section were found in LL and NL plants, the number increased to 10 in HL plants and was highest in NatL plants, with 12 plastoglobules per chloroplast cross-section.

### Chloroplast lipid composition is similar in plants from all growth light conditions

**Figure 4:**
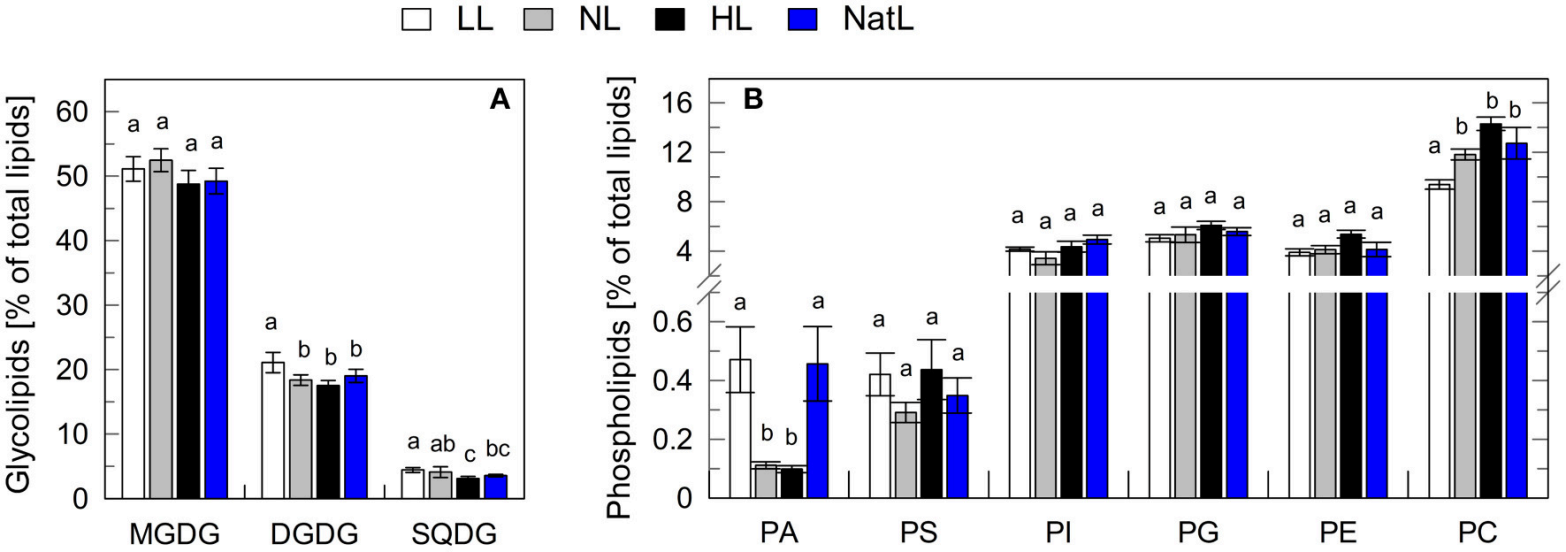
**Lipid composition of leaves. (A)** Relative amount of glycolipids. **(B)** Relative amount of phospholipids. MGDG, Monogalactosyldiacylglycerol; DGDG, Digalactosyldiacylglycerol; SQDG, Sulfoquinovosyldiacylglycerol; PA, Phosphatidic acid; PS, Phosphatidylserine; PI, Phosphatidylinositol; PG, phosphatidylglycerol; PE, and phosphatidylethanolamine; PC, Phosphatidylcholine. Mean values ± *SD* of five independent samples are shown. Significant differences [Holm-Sidak test, *p* < 0.05, for **(A)**; Dunn's test, *p* < 0.05, for **(B)**] are indicated.

Total leaf lipid extracts were further analyzed with respect to lipid classes and fatty acid composition. The relative contribution of glycolipids and phospholipids to the total amount of lipids was similar among all growth conditions (Figure 4). About 75% of the lipids in leaves were glycolipids (Figure 4A), with monogalactosyldiacylglycerol (MGDG, 50%) being the major constituent, followed by digalactosyldiacylglycerol (DGDG, 20%) and sulfoquinovosyldiacylglycerol (SQDG, 5%), in agreement with previous findings (Benson et al., 1959; Welti et al., 2002). The remaining 25% of membrane glycerolipids in leaves were phospholipids (Figure 4B), with phosphatidylcholine (PC) being the main constituent (12–16%). The amount of PC increased with increasing growth light intensity in LL, NL, and HL plants, and the PC content of NatL plants was similar to that of NL plants. In contrast, the amount of phosphatidic acid (PA) was highest in LL and NatL grown plants, while the amount of PS was significantly lower only in NL grown plants in comparison with LL and HL grown plants (Figure 4B). Also the saturation level of the fatty acids was very similar in the plants grown at different light regimes (Table S1). In conclusion, different growth light conditions do not have a pronounced impact on the lipid composition of the thylakoid membrane. Therefore, it is unlikely that the lipid composition is the key determinant for the observed differences in thylakoid membrane organization.

### The protein and pigment composition of thylakoid membranes from NatL plants share characteristics of LL and HL plants

To assess differences in the composition of the photosynthetic electron transport chain, the protein and pigment content of the thylakoid membrane was analyzed. No pronounced differences in the PSI and PSII content on Chl basis were determined among plants from different growth conditions (Table 2). In general, the amount of PSI (1.7–2 mmol per mol Chl) was slightly lower compared to that of PSII (2.0–2.5 mmol per mol Chl), resulting in PSII/PSI ratios of about 1–1.3. In NL plants, significantly more PSI was found in comparison to other growth light conditions, whereas PSII was most abundant in HL plants. NatL plants showed similar PSI amounts as LL and HL plants, but lower amounts of PSII than HL plants. In contrast, the amount of Cyt b_6_f varied strongly (in the range from 0.3 to 0.8 mmol Cyt b_6_f per mol Chl) and showed a positive correlation with increasing constant growth light intensity (HL>NL>LL). In HL plants, about 2–3 fold higher levels of Cyt b_6_f were determined compared to plants from other growth conditions (Table 2). This particular response of the Cyt b_6_f content to different growth light intensities has been reported before (Leong and Anderson, 1984), so that the low Cyt b_6_f content of NatL plants suggests a LL acclimated electron transport chain on basis of the abundance of protein complexes. In contrast, typical HL acclimation characteristics were determined for NatL plants with respect to the PsbS level and the xanthophyll cycle pigment pool (VAZ pool) size. Both the PsbS content and the VAZ pool size increased with increasing growth light intensities and highest levels were found in NatL plants (Table 2, Figure S2), in agreement with the literature (Mishra et al., 2012). Hence, NatL plants share properties of both LL and HL acclimated plants at the level of the protein and pigment composition of the thylakoid membrane.

**Table 2:** **Pigment and protein composition**.

**Parameter**	**Growth condition**			
	**LL**	**NL**	**HL**	**NatL**
Chl a/b	3.46 ± 0.12^a^	3.68 ± 0.18^b^	4.41 ± 0.23^c^	3.79 ± 0.26^ab^
β-carotene	55 ± 2^a^	61 ± 4^b^	63 ± 2^bc^	66 ± 2^c^
neoxanthin	34 ± 0^a^	32 ± 1^b^	32 ± 2^b^	31 ± 2^b^
VAZ pool	19 ± 1^a^	24 ± 3^b^	33 ± 3^c^	35 ± 6^c^
PSII	2.14 ± 0.14^ab^	1.99 ± 0.15^b^	2.51 ± 0.04^a^	1.86 ± 0.09^b^
PSI	1.76 ± 0.03^a^	2.09 ± 0.03^b^	1.87 ± 0.04^c^	1.80 ± 0.02^ac^
Cyt b_6_f	0.29 ± 0.05^a^	0.43 ± 0.04^a^	0.80 ± 0.04^b^	0.32 ± 0.03^a^
PsbS	0.74 ± 0.15^a^	1.00 ± 0.00^ab^	1.20 ± 0.34^bc^	1.34 ± 0.20^c^
PSII/PSI	1.22	0.95	1.34	1.03
PSII/Cyt b_6_f	7.38	4.63	3.14	5.81
PSI/Cyt b_6_f	6.07	4.86	2.34	5.63
PsbS/PSII (a.u.)	0.69	1.00	0.95	1.43

*The pigment composition of thylakoid membranes was derived from HPLC analyses. Carotenoid levels are given in mmol (mol Chl)^−1^. Mean values ± SD of at least 10 samples are shown. The amount of PSII, PSI, and Cytb_6_/f (expressed in mmol (mol Chl)^−1^) was determined from spectroscopic measurements of the respective activities. Mean values ± SD of 5 independent experiments are shown. PsbS levels were derived from Western blot analyses. The values represent relative amounts of PsbS normalized to the amount of NL plants. Mean values ± SD of five independent experiments are shown. For all parameters, significant differences (Holm-Sidak or Dunn's test, p < 0.05) are indicated by superscripted letters. The ratios of protein complexes were calculated from the respective mean values*.

### NatL plants combine light utilization characteristics of LL and HL plants

**Figure 5:**
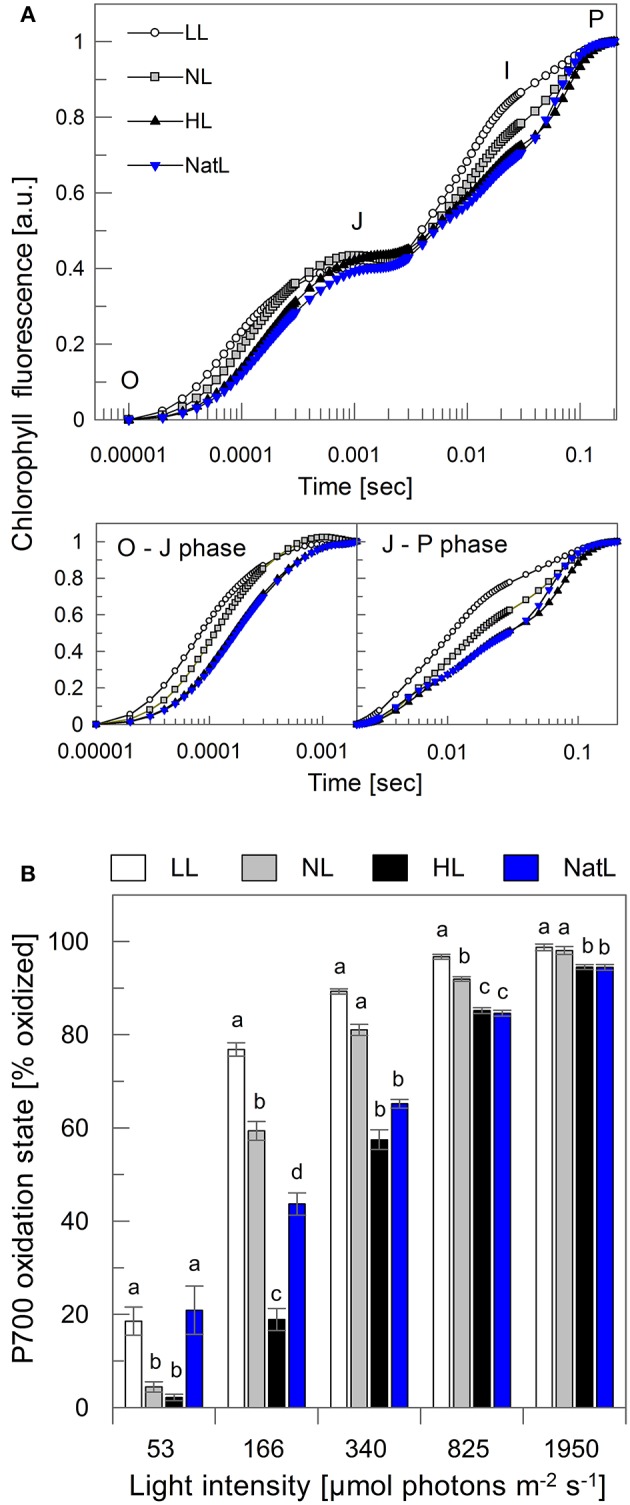
**Electron transfer from PSII to PSI. (A)** Chl fluorescence induction transients normalized to the total amplitude from the O to the P state (upper panel). The lower panel depicts the O to J phase (left) and the J to P phase (right), again normalized to the respective total amplitude. The normalization allows for direct comparison of the fluorescence induction kinetics. O, original fluorescence, corresponding to Fo; J and I, intermediate states; P, fluorescence peak, corresponding to Fm. Mean values of 10 measurements are shown, *SD* was <0.04. **(B)** P700 oxidation state at five different illumination intensities. Mean values ± SE of at least five independent samples are shown. Significant differences (Holm-Sidak or Dunn's test, *p* < 0.05) are indicated.

The time course of the fluorescence increase from Fo to Fm (OJIP transient) provides information about electron transfer between PSII and PSI (Stirbet and Govindjee, 2011). The O-J transient reflects the reduction of Q_A_ in PSII, the J-I phase the reduction of the plastoquinone (PQ) pool, and the I-P phase indicates the reduction of the acceptor side in PSI. As shown in Figure 5A, the overall fluorescence increase was fastest in LL plants. This was apparent for both, the intra-PSII electron transfer to Q_A_ (O-J phase) and the electron transfer to the PQ pool (J-I phase). Intermediate kinetics were found for NL plants, while HL and NatL plants showed slowest reduction of both Q_A_ (O-J) and PQ (J-I), although a distinct plateau of the I-phase was not clearly distinguishable from the J-P transient (Figure 5A).

The kinetics of Q_A_ reduction are known to reflect the functional antenna size of PSII (Malkin et al., 1981) with a larger antenna leading to a faster Q_A_ reduction. This suggests that LL plants possess the largest functional antenna, followed by NL plants and finally HL and NatL plants. This interpretation is in agreement with the determined Chl a/b ratios, which increased with increasing light intensities during growth at continuous light (Table 2). However, NatL showed a slow O-J increase (like HL plants) although the Chl a/b ratio was similar to NL plants, indicating that the antenna composition is not the only determinant for efficient Q_A_ reduction in PSII. Electron transfer was further monitored through measurements of the P700 oxidation state at different actinic light intensities (Figure 5B). The data determined for LL, NL, and HL plants revealed the expected differences of the P700 oxidation state. LL plants showed a high oxidation of P700 (of about 80%) at rather low light intensities of 166 μmol photons m^−2^ s^−1^, while similar oxidations states of P700 were reached only at higher light intensities in NL plants (at 340 μmol photons m^−2^ s^−1^) and HL plants (at 825 μmol photons m^−2^ s^−1^). Strikingly, NatL plants showed similar PSI oxidation states as LL plants at low actinic light intensities, but similar PSI oxidation states as HL plants at the two highest analyzed light intensities (Figure 5B). This suggests that NatL plants share properties of both LL and HL plants, and reflects the ability of NatL plants to cope efficiently with both low and high light intensities. It has been shown earlier, that the oxidation state of P700 is crucial for the photoprotection of PSI under high light (Tikkanen et al., 2014). To keep P700 in a partially oxidized state a low light might thus represent an advantage under rapidly fluctuating light conditions.

The light utilization capacity of the plants was further studied by Chl fluorescence and absorption spectroscopy under steady state conditions at the end of 30 min illumination at three different actinic light intensities (Table 3). At the level of electron transport and the fraction of oxidized Q_A_ (as reflected by the parameter qL), NatL plants showed properties of HL plants. The same held true for the NPQ capacity. However, NatL plants showed even a slightly higher capacity of pH-regulated qE quenching than HL plants, not only at the level of the maximum qE under light-saturated steady state conditions, but also for the transient qE under light-limiting conditions (Table 3). Differences in the maximum qE capacity maybe related to differences in the lumen pH, and/or the amount of PsbS or Zx. In fact, NatL plants showed slightly higher PsbS levels than HL plants (Table 2, Figure S2), but significantly lower Zx levels (Table 3). We assessed the lumen acidification by DIRK (dark interval relaxation kinetics) analysis of electrochromic absorption changes at 515 nm (Takizawa et al., 2007). The total proton motive force (*pmf*) (= ECS_total_) was found to be statistically not significantly different among all types of plants, but the fraction of the *pmf* stored as ΔpH (ECS_inv_) was highest in NatL plants and LL plants (Table 3). This indicates that the lumen pH is significantly lower in NatL and LL plants compared to NL and HL plants. The proton conductivity of the ATP synthase (gH^+^), however, was lower in LL plants (about 14–17 s^−1^) than in all other plants (about 23–34 s^−1^). Hence, also at the level of *pmf* partitioning and proton consumption by the ATP synthase, NatL plants combine properties of LL plants (*pmf* partitioning) and HL plants (proton consumption). The high qE capacity of Natl plants is therefore likely determined by combination of increased PsbS levels (as in HL plants) and a low lumen pH (as in LL plants).

**Table 3:** **Light utilization parameters at the end of 30 min illumination at three actinic light (AL) intensities: 340, 825, and 1950 μmol photons m^**−2**^ s^**−1**^ (μE)**.

**Parameter**	**AL [μE]**	**Growth condition**			
		**LL**	**NL**	**HL**	**NatL**
ETR [μmol m^−2^ s^−1^]	340	21 ± 1^a^	38 ± 2^a^	72 ± 4^b^	65 ± 3^b^
	825	43 ± 4^a^	44 ± 3^a^	84 ± 4^b^	81 ± 4^b^
	1950	117 ± 8^ab^	101 ± 8^b^	144 ± 10^a^	159 ± 13^a^
qL	340	0.063 ± 0.003^a^	0.111 ± 0.008^b^	0.291 ± 0.022^c^	0.258 ± 0.018^c^
	825	0.074 ± 0.009^a^	0.060 ± 0.004^a^	0.114 ± 0.005^b^	0.124 ± 0.006^b^
	1950	0.039 ± 0.005^a^	0.032 ± 0.002^a^	0.045 ± 0.007^a^	0.053 ± 0.011^a^
NPQ (qE)	340	1.03 ± 0.02^a^	1.21 ± 0.05^ab^	1.85 ± 0.12^bc^	2.15 ± 0.07^c^
	825	1.23 ± 0.02^a^	1.21 ± 0.06^a^	2.09 ± 0.07^b^	2.23 ± 0.15^b^
	1950	0.83 ± 0.11^a^	1.71 ± 0.05^b^	2.60 ± 0.10^c^	2.85 ± 0.09^c^
NPQ (qI)	340	0.40 ± 0.01^a^	0.23 ± 0.03^b^	0.20 ± 0.03^b^	0.22 ± 0.04^b^
	825	1.06 ± 0.04^a^	0.77 ± 0.03^b^	0.56 ± 0.04^c^	0.56 ± 0.07^c^
	1950	3.40 ± 0.13^a^	2.29 ± 0.16^b^	0.61 ± 0.08^c^	0.98 ± 0.20^c^
Transient NPQ	10	0.19 ± 0.03^a^	n.d.	n.d.	n.d.
	50	n.d.	0.34 ± 0.03^b^	0.39 ± 0.03^b^	0.68 ± 0.05^c^
Zx [% VAZ]	340	40 ± 1^a^	53 ± 1^b^	55 ± 6^b^	39 ± 1^a^
	825	35 ± 2^ab^	48 ± 2^b^	46 ± 4^ab^	31 ± 3^a^
	1950	42 ± 1^a^	50 ± 1^b^	61 ± 1^c^	40 ± 2^a^
ECS_total_	340	0.87 ± 0.08^a^	1.26 ± 0.05^b^	0.76 ± 0.06^a^	0.75 ± 0.07^a^
	825	0.76 ± 0.09^a^	0.82 ± 0.10^a^	0.75 ± 0.08^a^	0.66 ± 0.05^a^
	1950	0.72 ± 0.15^a^	0.76 ± 0.07^a^	0.77 ± 0.10^a^	0.57 ± 0.06^a^
ECS_inv_ [%]	340	45.1 ± 18.5^a^	39.0 ± 6.4^a^	42.9 ± 9.3^a^	49.3 ± 5.9^a^
	825	61.0 ± 19.8^a^	52.0 ± 9.8^a^	32.8 ± 12.3^b^	62.0 ± 11.1^a^
	1950	87.0 ± 7.1^a^	76.6 ± 12.7^a^	70.5 ± 14.4^a^	88.1 ± 4.5^a^
gH^+^ [s^−1^]	340	17.4 ± 3.6^a^	31.1 ± 5.5^ab^	31.5 ± 7.3^ab^	34.2 ± 4.3^b^
	825	14.1 ± 7.5^a^	23.8 ± 5.6^ab^	28.1 ± 3.0^b^	23.0 ± 0.9^ab^
	1950	13.9 ± 4.2^a^	28.9 ± 7.5^b^	35.1 ± 11.4^b^	27.4 ± 5.6^b^

*The electron transport rate (ETR), the fraction of oxidized Q_A_ (qL), and the NPQ parameters qE and qI were derived from Chl fluorescence analysis. The relative Zx content (in % of the total VAZ pool) was determined by HPLC analyses. The total pmf (ECS_total_), the fraction of the pmf stored as ΔpH (ECS_inv_) and the proton conductivity of the ATP synthase (gH^+^) was derived from DIRK (dark interval relaxation kinetics) analysis of electrochromic absorption changes. The maximum value of the transient NPQ was determined from Chl fluorescence quenching analyses at non-saturating actinic light intensities (10 μE for LL plants and 50 μE for NL, HL, and NatL plants). Mean values ± SD of 3–6 independent experiments are shown. For all parameters, significant differences (Holm-Sidak or Dunn's test, p < 0.05) are indicated by superscripted letters*.

### The high qE capacity of HL plants, but not of NatL plants, is based on energy transfer to PSI

**Figure 6:**
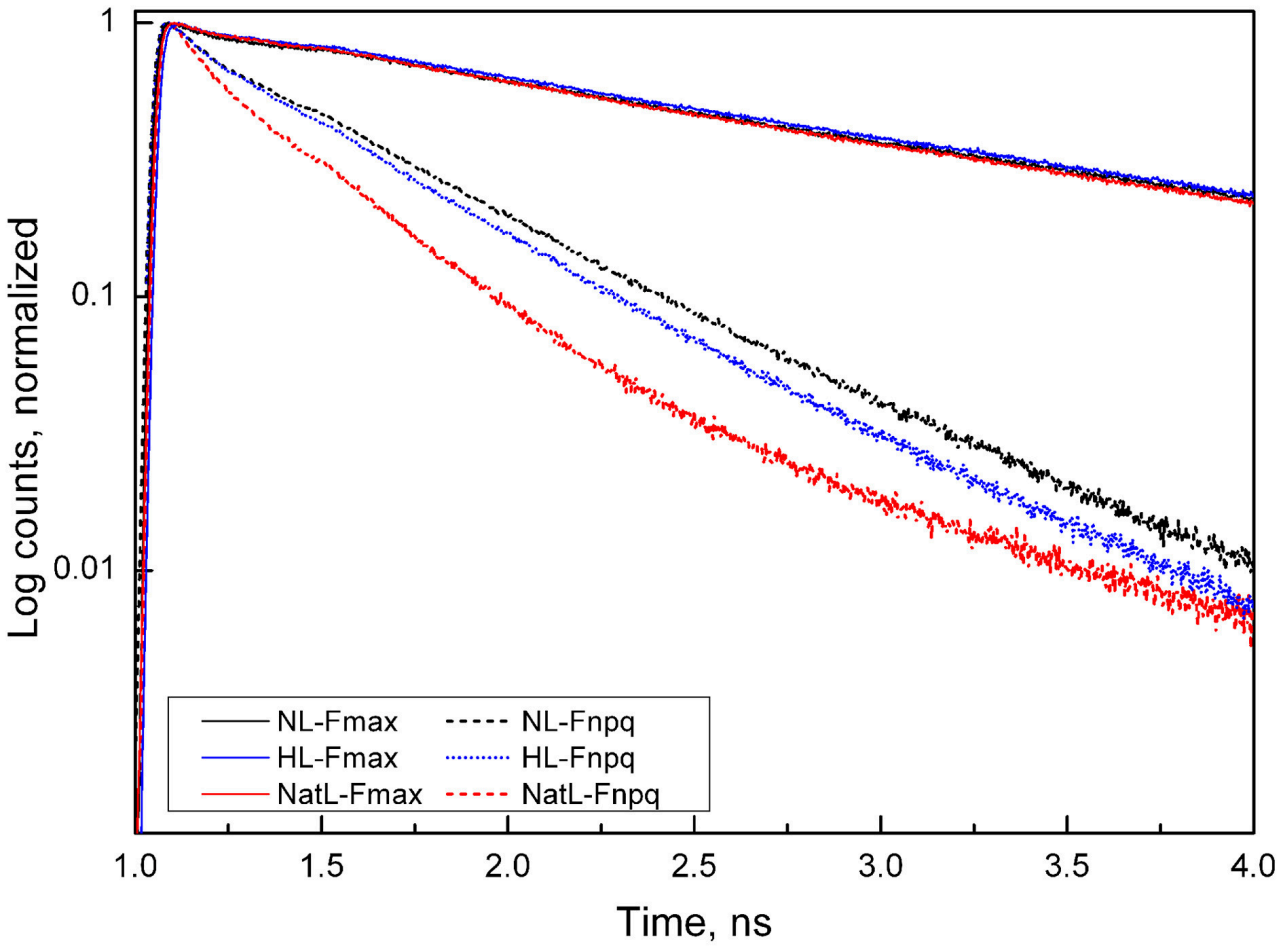
**Normalized fluorescence decays in Arabidopsis leaves**. Fluorescence decay was measured in either unquenched dark-acclimated state (Fmax) or in the quenched light-acclimated state (F_NPQ_). The excitation wavelength was 663 nm and the emissions measured at 683 nm. The faster decay in the F_NPQ_ state indicates the activation of fluorescence quenching. Note that plants grown under NatL conditions exhibited the strongest quenching.

We further investigated the underlying quenching mechanisms by ultrafast fluorescence measurements. It was not possible to perform these measurements with LL plants due to the small leaf size of these plants. Analysis of the fluorescence decay kinetics measured at 686 nm (Figure 6) revealed similar average lifetimes (τ_av_) of about 1.3 ns in the dark-adapted F_max_ state of NL, HL, and NatL plants (Table 4). The accelerated decay in the light-adapted state (F_NPQ_) reflects the NPQ induction of NPQ in all cases. In comparison with NL plants (τ_av_ = 380 ps), a slightly faster decay was found for HL plants (τ_av_ = 320 ps) and a much faster decay for NatL plants (τ_av_ = 130 ps), reflecting the most efficient quenching in NatL plants. These lifetimes corresponded to NPQ values of 2.6 (NL plants), 3.1 (HL plants), and 8.5 (NatL plants). For NL and HL plants, the NPQ values were somewhat higher but still similar to those derived from steady state fluorescence measurements (Table 3), while the NPQ value was much higher for NatL plants. This discrepancy is related to the fact that steady state NPQ values are determined from fluorescence emitted at wavelength >720 nm, while the NPQ values obtained from time-resolved measurements were derived from the fluorescence at 686 nm. Obviously, the NPQ at this PSII specific wavelength is much higher in the red region as compared to the far-red region.

**Table 4:** **Average lifetimes and rate constants of F_**max**_ and F_**NPQ**_ components in NL, HL and NatL grown ***A. thaliana*** plants**.

**Parameter**	**NL**	**HL**	**NatL**
τ_av_, ps at 686 nm (F_max_)	1,380	1,320	1,240
τ_av_, ps at 686 nm (F_NPQ_)	380	320	130
NPQ_total_ at 686 nm	2.6	3.1	8.5
k_D_max, PSII_, ns^−1^	0.3	0.3	0.3
k_D_NPQ, PSII_, ns^−1^	1.8	–	3.2
k_D_NPQ, PSII−C_, ns^−1^	–	1.6	–
k_D_LHCII_, ns^−1^	3.4	2.21	4.3
Lifetime τ [ps] of detached antenna component	292	453	233
Detached antenna cross-section (% of total PSII antenna)	30	40	30

*Upper part: Average lifetimes τ_av_ [ps] of the fluorescence decay measurements at 686 nm under F_max_ and F_NPQ_ conditions shown in Figure S3 and the corresponding NPQ values. Middle part: Rate constants k_D_ [ns^−1^] for PSII antenna deactivation of unconnected (PSII) and connected (PSII-C) PSII reaction centers under F_max_ and F_NPQ_ conditions. Lower part: Lifetime τ [ps] of the detached PSII antenna and the relative decrease of the PSII antenna cross-section (in% of the total PSII antenna). Errors in the values were in the range of ± 5–10%*.

Decay-associated spectra (DAS), which carry both, spectral and kinetic information, were derived from global and target analysis (van Stokkum et al., 2004; Holzwarth et al., 2009; Slavov et al., 2016; Figure S3). In Arabidopsis leaves, such analyses result in identification of 4 components related to PSI (lifetimes ranging from 4 to 100 ps) and at least 3 components related to PSII (lifetimes ranging from 35 ps to 2 ns; Holzwarth et al., 2009; Miloslavina et al., 2011). Analysis of the PSII related spectra allowed for determination of the rate constant k_*D*_, which represents the non-photochemical deactivation rate in the PSII-attached antenna and is thus a direct measure of NPQ (Table 4). NL plants showed an increase of k_D,PSII_ from 0.3 ns^−1^ in the dark-adapted state (k_D,max,PSII_) to 1.8 ns^−1^ in the light-adapted (k_D,NPQ,PSII_) state, in accordance with former studies (Holzwarth et al., 2009; Miloslavina et al., 2011). Moreover, also the light-induced detachment of a fraction of LHCII (k_D,LHCII_, Table 4), which gives rise to the PsbS-dependent activation of the quenching site Q1 (Holzwarth et al., 2009; Miloslavina et al., 2011), was detectable. Compared to that, NatL plants showed the same general features, but both k_D,LHCII_ and k_D,NPQ, PSII_ were higher than in NL plants, reflecting an increased quenching involving the PsbS-dependent Q1 site and the Zx-dependent Q2 site, respectively.

In contrast to NL and NatL plants, it was not possible to fit the data obtained with HL plants with the classical kinetic schemes for separated PSII and PSI centers (Holzwarth et al., 2009). Instead it was required to assume a heterogeneous PSII pool, with a fraction of PSII being connected to PSI (PSII-C) to obtain satisfying fits of the data, as has been described recently for HL acclimated microalgae (Slavov et al., 2016). This type of quenching is based on an energy transfer to PSI and requires a reorganization of the thylakoid membrane structure, by allowing direct contact between PSII and PSI, which is usually prevented by the heterogeneous distribution of PSII (in grana stacks) and PSI (in stroma lamellae). In addition to this quenching mechanism, also the PsbS-dependent Q1 site becomes activated by high light in HL plants (Table 4, Figure S3). However, the corresponding rate constant k_D,LHCII_ was lower than in NL and NatL plants, indicating a less efficient quenching in Q1 in HL plants.

In conclusion, the analyses of ultrafast fluorescence kinetics underline that the high NPQ capacities of HL and NatL plants are based on different mechanisms. In HL plants, energy transfer to PSI is the major NPQ mechanism, whereas more efficient quenching related to the Q1 and Q2 site is responsible for the high NPQ in NatL plants.

### Activation of qE in NatL plants involves rapidly reversible thylakoid membrane reorganization

**Figure 7:**
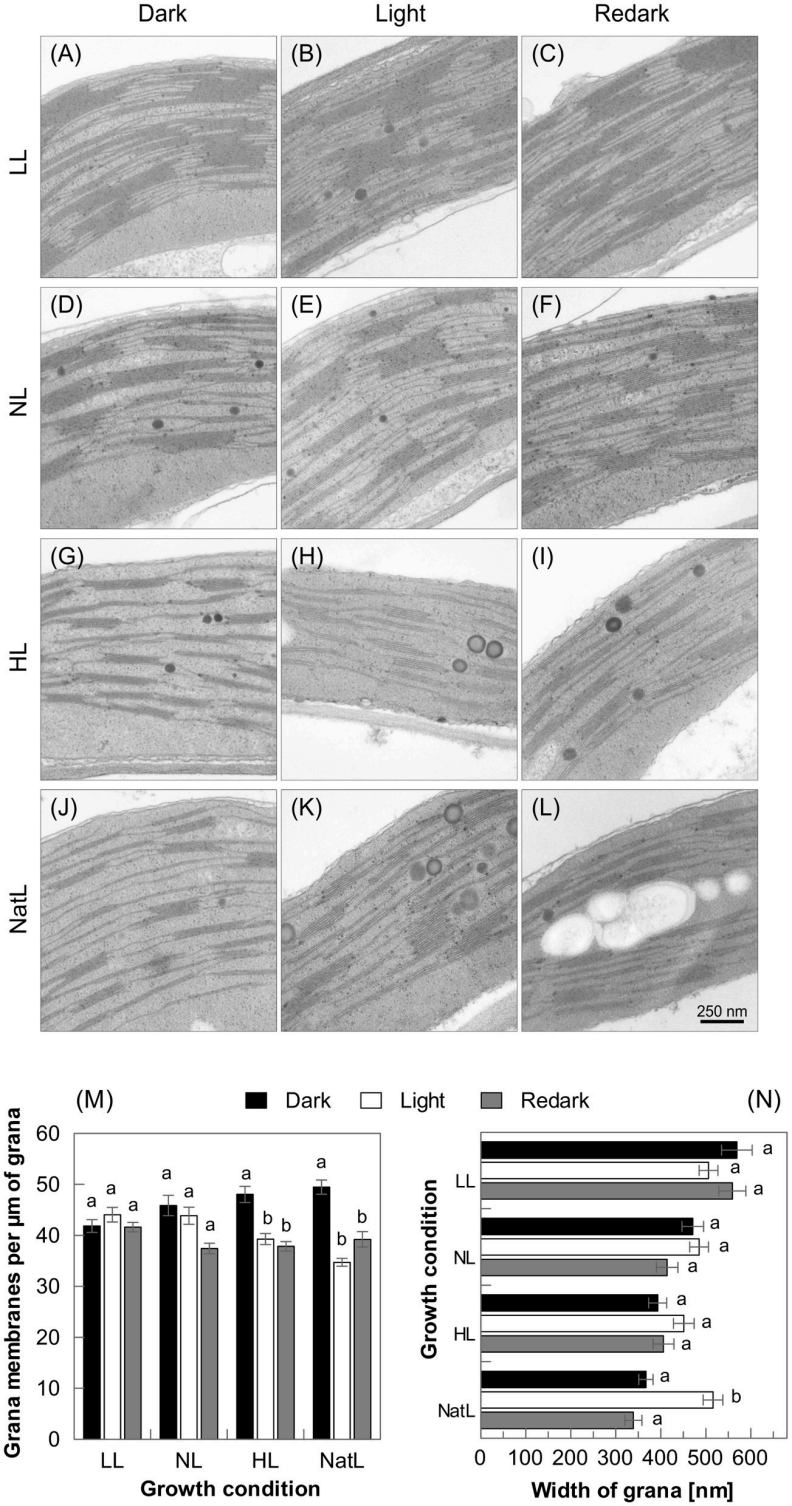
**Structural dynamics of thylakoid membrane stacking**. Upper panel: Transmission electron microscopic images of thylakoid membranes of LL **(A–C)**, NL **(D–F)**, HL **(G–I)**, and NatL **(J–L)** plants in the dark- and light acclimated state, and after 10 min of re- darkening. Lower panel: Quantitative analysis of **(M)** grana height and **(N)** grana width. 16–20 images were analyzed for each condition. The light-adapted state was induced by 30 min illumination with white light at an intensity of 1000 μmol photons m^−2^ s^−1^. Significant differences (Holm-Sidak test, *p* < 0.05) as compared to the respective dark-acclimated state are indicated.

To assess a possible rearrangement of the thylakoid membrane structure upon short-term acclimation to high light, electron microscopic images of chloroplasts from dark acclimated leaves were compared with those from light-acclimated leaves (30 min at 1,500 μmol photons m^−2^ s^−1^) and subsequently re-darkened (10 min) leaves (Figure 7). For LL plants (Figures 7A–C) and NL plants (Figures 7D–F), no significant changes in the membrane structure were detectable upon transition from the dark-acclimated to the light-acclimated state (Figures 7M,N). In contrast, HL plants (Figures 7G–I) exhibited significant unstacking of grana after 30 min of illumination, but the unstacking was not reversible within 10 min of re-darkening (Figure 7M). NatL plants (Figures 7J–L), however, showed both light-induced grana unstacking and restacking after 10 min of re-darkening (Figure 7M). The light-induced increase of the grana height was more pronounced in NatL than in HL plants and only NatL plants showed a significant broadening of the grana stacks by about 35% parallel to unstacking (Figure 7N). This broadening was fully reversible during 10 min of re-darkening while the changes in grana height were only partially reversible (Figures 7M,N), suggesting that tight packing of grana stacks as in LL and NL plants may prevent light-induced grana unstacking. Structural rearrangement of the membrane in response to high light might thus require a reduced degree of grana stacking in the dark-acclimated state, as observed in HL and NatL grown plants. However, more pronounced unstacking was observed in NatL plants, even though the grana stacks found in NatL plants were thicker compared to those in HL plants. Thus, growth under NatL conditions provides increased flexibility of thylakoid membranes.

## Discussion

### Limitations of the comparison of indoor and outdoor grown plants

The comparability of characteristics of field-grown plants with those grown under controlled lab conditions is limited by the unknown impact of other environmental factors than light. To minimize such uncertainties, NatL plants were grown in the same soil and pots as all other plants, and all plants were watered manually to accomplish similar soil humidity. Furthermore, all plants were grown under long-day conditions (14 h light under constant light conditions and between 14 and 16 h light under NatL condition) to minimize the possible impact of differences in the photoperiod. Moreover, NatL plants were grown in periods when the outside temperature was between 15 and 25°C, which was in the range of the temperature in the lab (20°C). Since the saturation level of fatty acids, which represents a reliable indicator of long-term temperature acclimation (Falcone et al., 2004) was similar in all plants (Table S1), it can be assumed that indoor and outdoor grown plants did not differ in the general long-term acclimation to temperature.

However, daily changes in the ambient temperature have direct effects on enzyme activities and diffusion dependent processes, which may have impact on the overall metabolism, from nutrient uptake to single biochemical reactions. Importantly, temperature fluctuations will further alter the vapor pressure deficit (VPD) at a given relative air humidity. Recent work has shown, that changes in the VPD related to a temperature change of about 10°C alter both the size and aperture of stomata, and thus have a pronounced impact on water use efficiency (Arve et al., 2017). However, experiments with rice plants revealed, that the impact of fluctuating temperature, CO_2_ and humidity has less impact on NPQ and ETR than fluctuating light (Yamori, 2016). Hence, fluctuating temperature, CO_2_ and humidity predominantly affect water use efficiency and carbon fixation in the Calvin Benson Bassham cycle rather than direct regulation of NPQ and chloroplast structure. In addition to the photoperiod, temperature is a key determinant of the flowering time (Song, 2016) and thus of the development of plants. In indoor grown Arabidopsis plants, increase of the temperature generally triggers earlier flowering (Blázquez et al., 2003). However, fluctuating temperature conditions as experienced by field-grown plants strongly reduces the control of flowering time by temperature, leaving the photoperiod as the major determinant of FT under field conditions (Song, 2016). We therefore assume that fluctuations in temperature, VPD, air humidity, and CO_2_ concentrations may mainly influence water use efficiency, biomass accumulation and development of NatL plants, but not the light utilization characteristics at the level of the electron transport chain. Moreover, the parameters studied here are known to be markedly influenced in response to changes of only the growth light intensity. Therefore, it is reasonable to assume that the different growth conditions used in this work provide a reliable basis for studying specific differences in acclimation to different growth light regimes.

### Upper and lower limits of growth light intensities determine the characteristics of NatL plants

Plants grown under constant LL and HL intensities showed the well-known differences in leaf morphology and photosynthetic capacities (for details and references see Introduction). This was apparent from leaf thickness and CO_2_ assimilation (Figure 1), and the morphological and functional characteristics of chloroplasts and thylakoid membranes (Figures 2–5). NatL plants received median light intensities similar to that of NL plants, but achieved combined LL and HL properties as obvious from the characteristics of the composition and function of the electron transport chain. We therefore conclude that the upper and lower limits of growth light intensities (Figure S1) rather than the mean light intensity determine the light acclimation characteristics of plants.

### Unique thylakoid membrane composition provides flexible light utilization in NatL plants

The acclimation of plants to fluctuating light intensities in the field requires a flexible adjustment of the photosynthetic capacity to cope with rapid changes of non-saturating and saturating light intensities. Our analyses show that NatL plants acquired properties of both HL plants (e.g., leaf morphology, CO_2_ assimilation, and electron transport rates) and LL plants (e.g., Chl a/b ratio, lumen acidification), but further developed unique features (e.g., thylakoid membrane organization and dynamics). Hence, NatL combine different characteristics to ensure highly flexible light utilization in response to fluctuating light intensities.

At the level of photosynthetic electron transport, the amount of the Cyt b_6_/f complex is supposed to be the major site of photosynthetic flux control, because plastoquinol oxidation is the rate-limiting step of electron transport (Schoettler and Toth, 2014). The low abundance of Cyt b_6_/f in NatL plants (Table 2) represents a typical characteristic of LL plants, in line with the high fraction of oxidized P700 at low actinic light intensities in both LL and NatL plants (Figure 5B). Furthermore, the ratios of PSII/Cyt b_6_f and PSI/Cyt b_6_f determined for NatL, also resembled the ratios of LL plants and were much higher than that of HL plants (Table 2). However, at high actinic light intensities, the P700 oxidation state (Figure 5B), the redox state of Q_A_ (parameter qL, Table 3) and the dynamics of the reduction of the electron transport chain (OJIP transients, Figure 5A) of NatL plants were similar to that of HL plants. Obviously, a low Cyt b_6_/f content is not necessarily the key determinant for the redox state of the photosynthetic electron transport chain and of the electron transport characteristics.

Alternatively, differences in the membrane fluidity or the protein/lipid ratio may account for the observed electron transport characteristics of NatL plants. The lipid composition is known to have strong impact on membrane fluidity (Los and Murata, 2004; Mullineaux and Kirchhoff, 2009), which in turn might affect electron transport characteristics (Berry and Bjorkman, 1980; Los et al., 2013). However, the relative amounts of glycolipids and phospholipids, as well as the saturation level of fatty acids did not reveal significant differences among the plants from different growth conditions (Figure 4, Table S1). The protein density and the supramolecular organization of photosystems are known to influence the mobility of small molecules and large protein supercomplexes (Kirchhoff, 2014). Indeed, the amount of lipids per photosystem was strongly reduced in chloroplasts from NatL and HL plants as compared to LL and NL plants (Table 5). It can thus be speculated that the higher protein density in NatL and HL plants is responsible for more efficient electron transport at high light intensities.

**Table 5:** **Amount of Chl, lipids and protein complexes per chloroplast**.

**Parameter**	**Growth condition**			
	**LL**	**NL**	**HL**	**NatL**
Chl (a+b) [fmol]	1.09 ± 0.18^ab^	1.15 ± 0.14^a^	0.93 ± 0.16^b^	0.71 ± 0.07^b^
Lipids [fmol]	15.1	17.7	8.6	11.7
PSII [amol]	1.96	1.73	2.70	2.62
PSI [amol]	1.61	1.82	2.01	2.54
Cyt b_6_/f [amol]	0.27	0.37	0.86	0.45
Lipids/PSII	7.675	10.213	3.192	4.482
Lipids/PSI	9.332	9.724	4.285	4.632
Lipids/Cyt b_6_/f	56.638	47.265	10.015	26.053

*The values for the amount of Chl (a+b) ± SD were taken from Figure 2H. Superscripted letters indicate significant differences (Holm-Sidak or Dunn's test, p < 0.05). All other values were calculated from the respective mean values determined in relation to Chl*.

### Light-regulated thylakoid membrane dynamics provide a high NPQ capacity of NatL plants

At the morphological level, NatL exhibited specific characteristics with respect to the thylakoid membrane structure (Figure 3) and its light-dependent dynamics in the short-term (Figure 7). The thylakoid membrane structure of NatL plants was similar to that of HL plants, but more heterogeneous with respect to the number of membranes per granum (Figure 3). Moreover, chloroplasts from NatL plants contained the highest number of plastoglobules (Figure 3). Plastoglobules are lipid bodies in chloroplasts (Lichtenthaler, 1968), which are in physical contact with the thylakoid membrane in stroma exposed regions and supposed to function in lipid storage and biosynthesis (Austin et al., 2006). The number and size of plastoglobules varies not only during plant development and plastid differentiation, but also under oxidative stress conditions (Rottet et al., 2015). Hence, the observed differences in the plastoglobule content likely represent different levels of acclimation to photo-oxidative stress.

Another specific feature of NatL plants was the rapidly light-inducible (with 30 min) and dark-reversible (within 10 min) switch in the grana structure (Figure 7). The light-induced increase of the grana width was only detectable in NatL plants and occurred in parallel to the activation of NPQ. Similar changes have been described recently for sunlight grown Monstera plants (Demmig-Adams et al., 2015), which are also characterized by a high NPQ capacity. A high light-regulated flexibility of the thylakoid membrane might thus represent a key factor for the high NPQ of NatL grown plants. These structural changes might be promoted by high levels of PsbS and VAZ pool size (Table 2), which both control not only the NPQ capacity, but are further supposed to modify the membrane flexibility (Horton, 2014) and the structural arrangement of PSII supercomplexes (Ruban and Johnson, 2015). The high NPQ capacity of NatL plants was based on the known PsbS and Zx dependent quenching sites Q1 and Q2, while energy transfer from PSII to PSI was involved in the NPQ of HL plants (Table 4). The latter mechanism has recently been characterized in microalgae after exposure to extreme HL stress (Slavov et al., 2016). We speculate that such an energy spillover NPQ mechanism may become generally activated during exposure to constant HL conditions. However, the high NPQ capacity of both HL and NatL plants was accompanied by unstacking of thylakoid membranes (Figure 7). Unstacking of the membranes is supposed to indispensable for a spillover mechanism to allow for the required contact of PSII and PSI (Slavov et al., 2016). Our data suggest that the structural flexibility of the thylakoid membrane provides the basis for a high NPQ capacity of plants, independent of the underlying mechanism. It remains to be elucidated, which factors determine and regulate this structural flexibility. Our data indicate that a reduced degree of grana stacking, an increased fraction of stroma exposed membranes, and a low lipid/protein ratio might be a prerequisite for the structural flexibility. The rapid reversibility of unstacking observed in NatL plants points to a possible role of the transthylakoid pH gradient or light-regulated ion fluxes in the regulation of the structural dynamics.

In conclusion, our work shows that NatL plants exhibit a number of striking features that allow for optimal light utilization, and underlines the importance of the use of plants grown under NatL conditions for studying light acclimation in plants.

## Author contributions

PJ and TS wrote the manuscript, planned and designed the experiments. TS, SP, MM, and PD performed the experiments. All authors analyzed and interpreted the data; read and approved the final version of the manuscript.

## Funding

This work was supported by the Deutsche Forschungsgemeinschaft (DFG) to PJ (JA 665/9-1).

### Conflict of interest statement

The authors declare that the research was conducted in the absence of any commercial or financial relationships that could be construed as a potential conflict of interest.
