# Supplementary material for: Plant Growth under Natural Light Conditions Provides Highly Flexible Short-Term Acclimation Properties toward High Light Stress
Source: Front Plant Sci. 2017 May 3;8:681. doi: 10.3389/fpls.2017.00681 (PMC5413563; doi:10.3389/fpls.2017.00681)
Supplement: Supplementary file 1 [file Table1.PDF]

Supplementary Material

Plant growth under natural light conditions provides highly flexible short-term acclimation properties towards high light stress

Tobias Schumann, Suman Paul, Michael Melzer, Peter Dörmann, Peter Jahns*

*** Correspondence:** Peter Jahns: pjahns@hhu.de

Table S1 Molecular species composition of glycerolipids. The relative content of molecular species is given in mol % for each of the three lipid classes (MGDG, DGDG and PA). Mean values +/- SD of five independent samples are shown. MGDG: Monogalactosyldiacylglycerol; DGDG: Digalactosyldiacylglycerol; PA: Phosphatidic acid.

Lipid class	Fatty acids	Growth condition			
		LL	NL	HL	NatL
MGDG	34:6	70.82 ± 1.34	71.50 ± 0.45	76.61 ± 1.35	75.60 ± 2.00
	34:5	3.18 ± 0.27	1.82 ± 0.36	0.98 ± 0.26	0.00 ± 0.00
	34:4	2.72 ± 0.30	1.92 ± 0.09	1.22 ± 0.17	0.33 ± 0.05
	34:3	1.30 ± 0.10	0.98 ± 0.05	0.60 ± 0.09	0.23 ± 0.05
	34:2	0.69 ± 0.11	0.41 ± 0.02	0.22 ± 0.04	0.04 ± 0.02
	34:1	0.34 ±0.04	0.25 ± 0.03	0.13 ± 0.04	0.04 ± 0.01
	36:6	17.21 ± 0.83	21.00 ± 0.52	19.01 ± 1.18	20.62 ± 1.53
	36:5	1.69 ± 0.09	1.07 ± 0.06	0.60 ± 0.10	0.56 ± 0.20
	36:4	0.80 ± 0.13	0.60 ± 0.01	0.37 ± 0.06	0.66 ± 0.12
	36:3	0.12 ± 0.02	0.12 ± 0.03	0.06 ± 0.01	0.06 ± 0.01
	36:2	0.07 ± 0.02	0.03 ± 0.00	0.02 ± 0.01	0.02 ± 0.01
	36:1	0.07 ± 0.03	0.06 ± 0.01	0.05 ± 0.01	0.05 ± 0.01
	38:6	0.75 ± 0.53	0.13 ± 0.04	0.07 ± 0.02	1.50 ± 0.93
	38:5	0.07 ± 0.01	0.03 ± 0.01	0.03 ± 0.00	0.12 ± 0.03
	38:4	0.12 ± 0.02	0.07 ± 0.01	0.03 ± 0.00	0.16 ± 0.02
	38:3	0.05 ± 0.01	0.01 ± 0.01	0.01 ± 0.01	0.02 ± 0.01
DGDG	34:6	6.27 ± 0.80	7.00 ± 0.37	7.68 ± 0.39	6.53 ± 0.51
	34:5	1.71 ± 0.24	1.27 ± 0.13	0.96 ± 0.15	0.94 ± 0.19
	34:4	1.27 ± 0.10	0.87 ± 0.10	0.85 ± 0.13	0.53 ± 0.10
	34:3	12.70 ± 1.26	12.51 ± 0.65	15.38 ± 1.28	10.33 ± 1.00
	34:2	3.33 ± 0.27	2.07 ± 0.03	1.87 ± 0.34	1.08 ± 0.21
	34:1	1.62 ± 0.40	1.12 ± 0.13	1.01 ± 0.28	0.38 ± 0.10
	36:6	63.02 ± 2.56	67.57 ± 0.95	66.48 ±1.41	72.43 ± 0.65
	36:5	3.53 ± 0.67	2.18 ± 0.54	1.26 ± 0.59	0.85 ± 0.24
	36:4	1.24 ± 0.23	1.60 ± 0.31	1.45 ± 0.20	1.03 ± 0.30
	36:3	1.07 ± 0.18	1.09 ± 0.18	0.85 ± 0.18	0.52 ± 0.12
	36:2	0.27 ± 0.06	0.21 ± 0.08	0.09 ± 0.07	0.13 ± 0.07
	36:1	0.48 ± 0.13	0.49 ± 0.13	0.47 ± 0.22	0.37 ± 0.07
	38:6	2.70 ± 1.25	1.46 ± 0.25	1.26 ± 0.41	4.02 ± 1.57
	38:5	0.32 ± 0.19	0.25 ± 0.17	0.20 ± 0.08	0.29 ± 0.12
	38:4	0.38 ± 0.12	0.19 ± 0.09	0.08 ± 0.05	0.50 ± 0.10
	38:3	0.09 ± 0.07	0.14 ± 0.11	0.12 ± 0.07	0.08 ± 0.04
PA	34:6	1.08 ± 0.74	5.58 ± 2.56	3.16 ± 2.85	1.18 ± 0.56
	34:5	1.34 ± 0.94	0.95 ± 0.90	3.29 ± 3.79	0.72 ± 0.58
	34:4	1.17 ± 0.69	1.07 ± 1.65	1.90 ± 2.42	2.51 ± 1.15
	34:3	26.19 ± 5.78	16.53 ± 3.06	18.78 ± 8.01	22.25 ± 2.41
	34:2	16.73 ± 4.10	12.33 ± 5.00	18.06 ±3.59	12.45 ± 2.69
	34:1	3.03 ± 1.30	3.39 ± 2.13	2.72 ±3.25	1.39 ± 1.38
	36:6	12.46 ± 2.52	14.20 ± 5.45	14.94 ± 4.02	18.63 ± 3.55
	36:5	18.43 ± 3.05	18.64 ± 8.32	12.78 ± 3.83	23.03 ± 1.39
	36:4	13.64 ± 4.48	9.99 ± 2.61	11.62 ± 8.76	12.26 ± 3.86
	36:3	4.39 ± 1.36	13.58 ± 4.58	8.56 ± 5.72	3.69 ± 2.36
	36:2	1.55 ± 1.27	3.74 ± 4.45	4.19 ± 2.68	1.87 ± 1.31
