# Supplementary material for: Plant Growth under Natural Light Conditions Provides Highly Flexible Short-Term Acclimation Properties toward High Light Stress
Source: Front Plant Sci. 2017 May 3;8:681. doi: 10.3389/fpls.2017.00681 (PMC5413563; doi:10.3389/fpls.2017.00681)

Supplementary Material

Plant growth under natural light conditions provides highly flexible short-term acclimation properties towards high light stress

Tobias Schumann, Suman Paul, Michael Melzer, Peter Dörmann, Peter Jahns*

*** Correspondence:** Peter Jahns: pjahns@hhu.de

Figure S1 Light intensities during growth of NatL plants. Typical course of the light intensities during one month of the growing season, measured with a light sensor located between the plant pots. Light intensities are shown for each day in 3 h intervals between 6 am and 6 pm, exemplarily for the period from June 3 (day 1) till July 1 (day 30) in the year 2014.

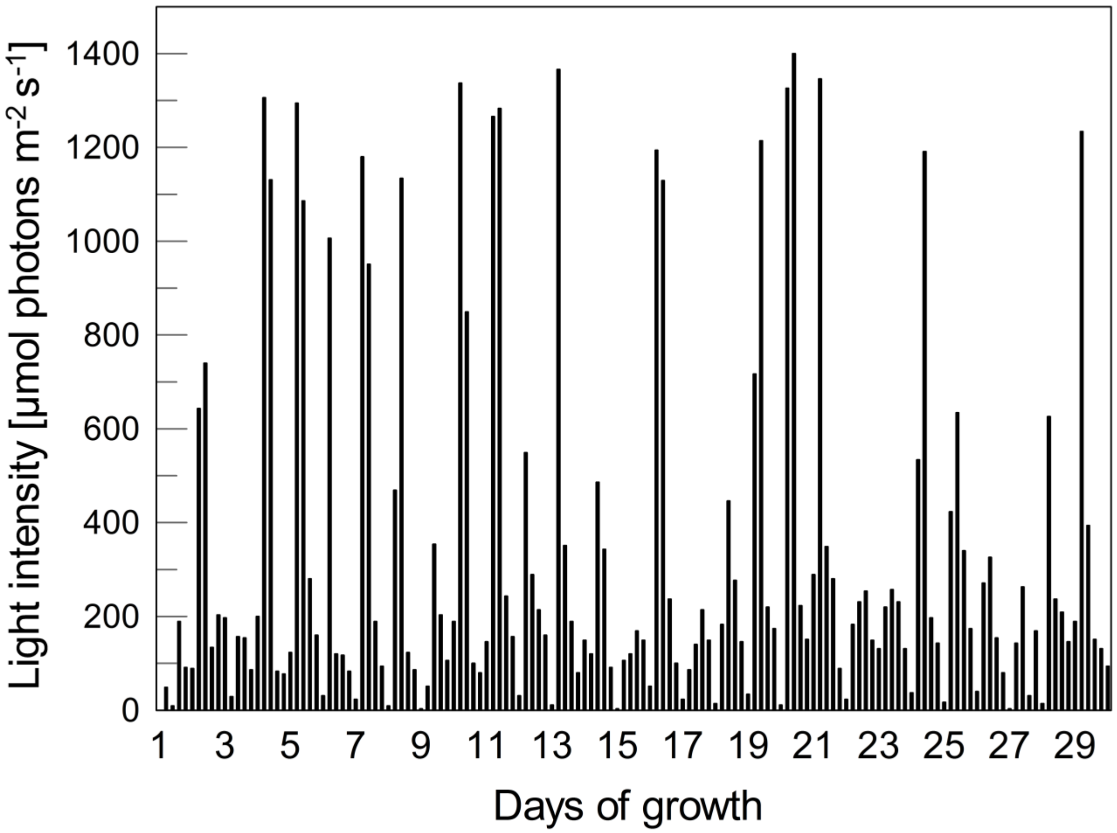

Supplement: Supplementary file 2 [file Image1.PDF]
