# Supplementary material for: Plant Growth under Natural Light Conditions Provides Highly Flexible Short-Term Acclimation Properties toward High Light Stress
Source: Front Plant Sci. 2017 May 3;8:681. doi: 10.3389/fpls.2017.00681 (PMC5413563; doi:10.3389/fpls.2017.00681)

Supplementary Material

Plant growth under natural light conditions provides highly flexible short-term acclimation properties towards high light stress

Tobias Schumann, Suman Paul, Michael Melzer, Peter Dörmann, Peter Jahns*

*** Correspondence:** Peter Jahns: pjahns@hhu.de

Figure S2 Quantification of the PsbS protein. For each growth condition, three different amounts (12, 10 and 8 µg) of total protein extract (as indicated) were loaded. The band intensity was quantified as described in the methods section. **(A)** Representative Western blot used for the quantification. **(B)** Quantitative analysis of the band intensities from five independent experiments. Significant differences (Dunn's test, p < 0,05) are indicated.

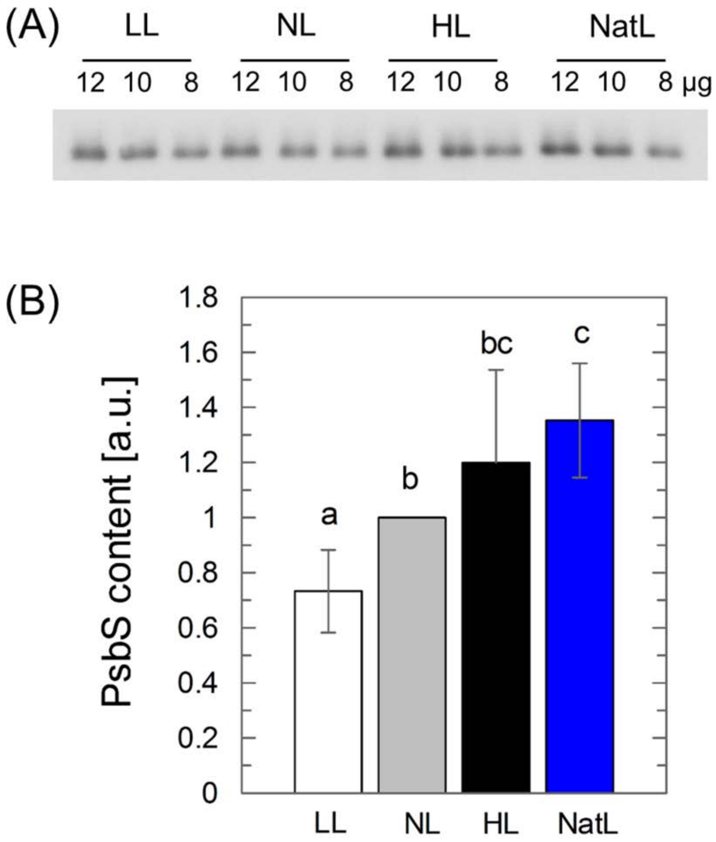

Supplement: Supplementary file 3 [file Image2.PDF]
