# Supplementary material for: Plant Growth under Natural Light Conditions Provides Highly Flexible Short-Term Acclimation Properties toward High Light Stress
Source: Front Plant Sci. 2017 May 3;8:681. doi: 10.3389/fpls.2017.00681 (PMC5413563; doi:10.3389/fpls.2017.00681)
Supplement: Supplementary file 4 [file Image3.PDF]

Supplementary Material

Plant growth under natural light conditions provides highly flexible short-term acclimation properties towards high light stress

Tobias Schumann, Suman Paul, Michael Melzer, Peter Dörmann, Peter Jahns*

*** Correspondence:** Peter Jahns: pjahns@hhu.de

Figure S3 Analysis of fluorescence lifetime components. Decay-associated spectra as obtained from global target analysis (Holzwarth et al., 2009) of the fluorescence decay of dark-adapted **(A,C,E)** and light-adapted **(B,D,F)** leaves from NL **(A,B)**, HL **(C,D)** and NatL **(E,F)** plants. For clarity, only the positive amplitudes are shown. PSI components are shown in dotted lines, PSII related spectra in solid lines. The rate constants for each component are indicated in each panel.

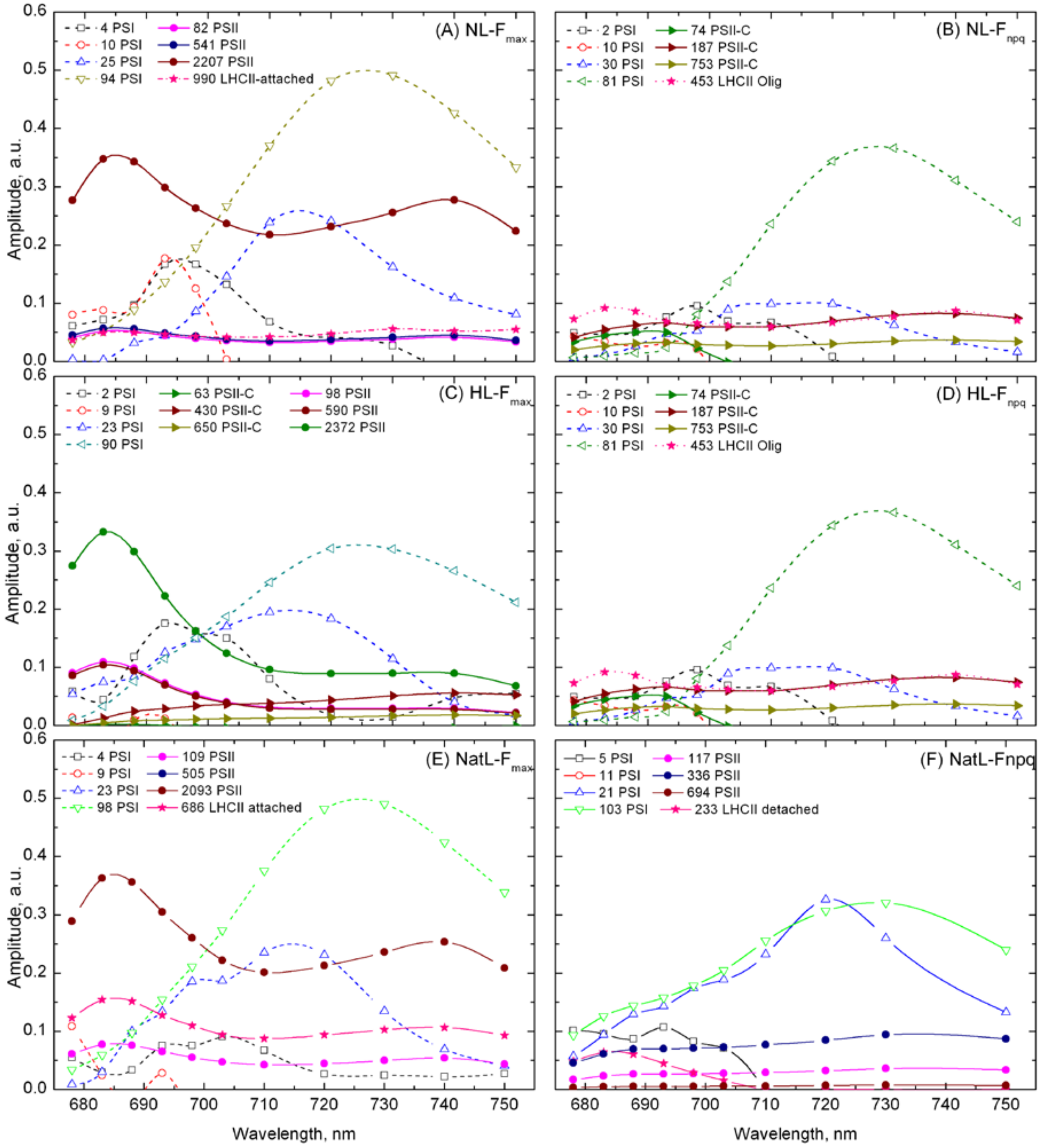

Reference

Holzwarth, A.R., Miloslavina, Y., Nilkens, M., and Jahns, P. (2009). Identification of two quenching sites active in the regulation of photosynthetic light-harvesting studied by time-resolved fluorescence. *Chem. Phys. Lett.* 483, 262-267.
